# Transdiagnostic Neurobiological and Nutritional Factors in Eating Disorders: Implications for Integrative Treatment Models

**DOI:** 10.3390/nu18071108

**Published:** 2026-03-30

**Authors:** Izabela Łucka, Ariadna Dobrzańska, Jolanta Góral-Półrola, Patrycja Leśnicka, Marta Kopańska

**Affiliations:** 1 Department of Developmental Psychiatry, Psychotic Disorders and Old Age Psychiatry, Medical University of Gdansk, 80-210 Gdansk, Polandadobrzanska@icloud.com (A.D.); 2 Faculty of Pedagogy and Psychology, Jan Kochanowski University of Kielce, 25-029 Kielce, Poland; jolagoralpolrola@gmail.com; 3 Department of Medical Communication and Professional Competency Development, Faculty of Medicine, University of Rzeszów, 35-959 Rzeszów, Poland

**Keywords:** transdiagnostic factors, neurobiology, serotonin, leptin, ghrelin, HPA axis, micronutrients, integrative treatment

## Abstract

Eating disorders (EDs), including anorexia nervosa (AN), bulimia nervosa (BN), and binge-eating disorder (BED), are complex psychiatric conditions characterized by high morbidity and mortality. Increasing evidence suggests that beyond disorder-specific symptomatology, shared transdiagnostic mechanisms contribute to their onset and persistence. This narrative review synthesizes current data on neurobiological and nutritional factors implicated in EDs, with particular emphasis on trait–state interactions and starvation-induced neuroadaptations. Predisposing vulnerabilities such as heightened anxiety, cognitive rigidity, and perfectionism appear to interact with state-dependent biological alterations induced by malnutrition. Chronic dietary restriction is associated with measurable alterations in serotonergic and dopaminergic systems, altered reward processing, and persistent activation of the hypothalamic–pituitary–adrenal (HPA) axis. Experimental studies suggest that acute tryptophan depletion may transiently reduce anxiety in individuals with anorexia nervosa, suggesting that, in some individuals, food restriction may function as a biologically reinforced strategy of affect regulation. Furthermore, disturbances in leptin and ghrelin signaling, along with widespread micronutrient deficiencies—including zinc, iron, selenium, and B vitamins—may exacerbate cognitive inflexibility, mood instability, and impaired decision-making. These metabolic and endocrine adaptations may contribute to a self-perpetuating cycle in which starvation-induced neurochemical changes reinforce restrictive or dysregulated eating behaviors. Importantly, several of these mechanisms extend beyond anorexia nervosa and may represent common transdiagnostic processes across eating disorders and related mental health conditions, including anxiety, depression, and addictive behaviors. Recognition of these biological and nutritional factors has significant implications for treatment. Nutritional rehabilitation should be conceptualized not solely as weight restoration, but as a neurobiological recalibration of stress regulation, reward sensitivity, and affective processing systems. An integrative treatment approach that combines behavioral stabilization with attention to underlying neurobiological and relational mechanisms may offer a more comprehensive framework for long-term recovery.

## 1. Introduction

Eating disorders (EDs), including anorexia nervosa (AN), emerge within complex personality, relational, systemic, and transgenerational contexts. Their psychopathology cannot be adequately understood through symptom-focused models alone, nor fully captured by highly structured and protocol-driven therapeutic approaches. While manualized treatments have contributed significantly to evidence-based practice, they do not always encompass the multilayered psychological and biological processes that maintain these conditions.

This paper integrates clinical reflection with a narrative review of empirical findings on cognitive behavioral therapy for eating disorders (CBT-ED), with particular attention to the enhanced CBT model (CBT-E) developed by Fairburn [1,2]. The central question concerns the conditions under which protocol-driven interventions genuinely support recovery and when they may inadvertently reinforce psychopathology—by sustaining the centrality of weight and control, reinforcing perfectionistic standards, or overlooking the symptom’s regulatory and relational functions.

Although eating-related behaviors constitute the most visible axis of pathology, they represent only one dimension of a broader disturbance involving identity organization, affect regulation, attachment patterns, and biological vulnerability. Clinically, the illness frequently serves an organizing function: it may regulate overwhelming affect, stabilize a fragile self-structure, or function as a relational language in systems where emotional communication is constrained.

In this context, anorexia is rarely reducible to a maladaptive habit or a simple consequence of distorted cognitions. Rather, it often operates as a “compromise formation”: psychologically costly and somatically destructive, yet subjectively necessary. The symptom may preserve internal cohesion, regulate interpersonal distance, or maintain systemic equilibrium. Consequently, resistance to recovery—conceptualized in CBT terms as avoidance—may also reflect unconscious efforts to preserve psychological integrity, particularly in relation to attachment within the therapeutic relationship [3,4,5].

Clinical experience suggests that standardized CBT protocols, though methodologically robust, do not always address critical dimensions of the disorder: the function of the symptom, dynamics of shame and control, ambivalence toward change, and family-level processes. In anorexia especially, sustaining mechanisms often extend beyond discrete core beliefs and are embedded in broader personality organization shaped by early attachment experiences and implicit transgenerational transmission.

Recognizing these limitations does not invalidate CBT-based models. Rather, it highlights the need for integrative approaches that combine empirically supported techniques with deeper understanding of relational, developmental, and biological mechanisms. Treatment guidelines may therefore benefit from incorporating individualized formulations that consider both trait-based vulnerabilities and state-dependent neurobiological adaptations.

This integrative perspective forms the foundation for the subsequent sections, which move from clinical conceptualization toward biological and transdiagnostic models of maintenance.

## 2. Methods (Narrative Review Approach)

This narrative review aims to synthesize current evidence on neurobiological and nutritional mechanisms involved in eating disorders (EDs), with a focus on trait–state interactions and starvation-induced neuroadaptations.

A literature search was performed using the following electronic databases: PubMed, Scopus, and Web of Science. The search covered studies published between January 2000 and January 2025. Keywords included combinations of: “eating disorders”, “anorexia nervosa”, “bulimia nervosa”, “binge-eating disorder”, “neurobiology”, “serotonin”, “dopamine”, “HPA axis”, “leptin”, “ghrelin”, “micronutrients”, and “nutritional rehabilitation”.

Studies were selected based on relevance to neurobiological, endocrine, and nutritional mechanisms in EDs. Priority was given to peer-reviewed empirical studies, meta-analyses, and systematic reviews. Both human and, where informative, animal studies were considered.

Exclusion criteria included non-peer-reviewed publications, single case reports, and studies not directly related to neurobiological or nutritional processes in EDs.

Given the narrative nature of the review, evidence was interpreted qualitatively, with attention to study design, sample characteristics (e.g., underweight vs. weight-restored), and potential confounding factors such as malnutrition, comorbidity, and medication use.

Studies were not formally weighted; however, greater interpretative emphasis was placed on findings supported by converging evidence across multiple methodologies (e.g., neuroimaging, endocrine, and behavioral studies).

## 3. Eating Disorders as a Clinical Phenomenon

Eating disorders belong to the most severe psychiatric conditions, not only due to profound psychological impairment but also because of potentially life-threatening somatic consequences. Mortality rates in anorexia nervosa remain among the highest in psychiatry [6].

Historically, EDs have been conceptualized within diverse theoretical paradigms, reflecting evolving understandings of the relationship between body, mind, and social context. Early descriptions emphasized symbolic and relational meanings of food refusal; fasting was interpreted as a pursuit of purity and control [7]. Such interpretations remain clinically relevant: refusal to eat is rarely a purely behavioral phenomenon but often carries layered psychological significance.

In psychoanalytic frameworks, anorexia has been understood as an expression of intrapsychic conflict, disturbances in object relations, separation difficulties, and dysregulated drive organization [8,9,10,11,12,13,14]. Systemic approaches conceptualize the disorder as embedded within family-level dynamics, where the symptom may arrest developmental transitions, stabilize alliances, or prevent overt conflict [15,16]. Across models, a shared insight persists: the symptom communicates.

Contemporary perspectives increasingly integrate biological vulnerability with psychological and systemic processes. Genetic predispositions and temperament traits interact with environmental stressors to produce heterogeneous clinical presentations [17,18]. Consequently, EDs should not be conceptualized as homogeneous diagnostic entities but as dynamic configurations of biological, psychological, and social vulnerabilities manifesting through similar behavioral patterns.

Diagnostic systems such as DSM-5 and ICD-10 provide valuable nosological clarity [19,20], yet clinical practice reveals fluidity between subtypes. Restrictive anorexia may evolve into binge–purge patterns; overt symptoms may remit while compensatory cognitive control intensifies. Symptom variability reflects underlying regulatory processes rather than discrete, static categories.

This diagnostic fluidity complicates strictly protocol-driven interventions. CBT models typically presuppose relatively stable symptomatic targets and linear treatment pathways. In reality, the symptom may shift form while preserving its regulatory function. Exclusive focus on current eating behavior risks addressing successive symptomatic manifestations without modifying core maintenance mechanisms.

Thus, eating disorders must be understood as dynamic, multi-level phenomena in which symptom expression represents the interface of personality organization, relational context, and biological vulnerability.

## 4. A Multifactorial Model: Neurobiology, Personality, and Relationships

Contemporary research supports a multifactorial model in which eating disorders arise from interactions between trait-based vulnerabilities and environmental influences [21,22]. Genetic susceptibility increases sensitivity to stressors rather than directly determining illness. Neurobiological research identifies dysregulation in stress systems, neurotransmitter functioning, and neurocognitive processes such as rigidity and harm avoidance.

### 4.1. Trait Vulnerabilities and Neurotransmitter Systems

Anorexia nervosa is increasingly conceptualized as involving trait-related alterations in serotonergic and dopaminergic systems [21,23]. Elevated serotonergic activity—particularly involving 5-HT1A and 5-HT2A receptors—has been associated with heightened anxiety, perfectionism, and behavioral inhibition [21]. These characteristics often precede illness onset, suggesting trait vulnerability.

Paradoxically, dietary restriction reduces tryptophan availability and consequently decreases central serotonin synthesis. This starvation may in some individuals be experienced as anxiolytic [3,24], transforming restriction into a form of maladaptive affect regulation. In this framework, starvation becomes biologically reinforced through negative reinforcement mechanisms.

Dopaminergic abnormalities within mesolimbic circuits further complicate reward processing. Food-related stimuli may elicit anxiety rather than pleasure, while weight loss and excessive control activate reward pathways [25,26]. Such paradoxical reinforcement explains the persistence of self-destructive behaviors despite medical risk.

### 4.2. HPA Axis and Stress Reactivity

Hyperactivation of the hypothalamic–pituitary–adrenal (HPA) axis and elevated cortisol levels are consistently documented in anorexia nervosa [22]. Chronic hypercortisolemia contributes to hypervigilance, anticipatory anxiety, and difficulty extinguishing stress responses. Clinically, this manifests as intolerance of uncertainty and heightened reactivity to minor environmental changes.

Importantly, these starvation-induced adaptations may amplify pre-existing traits such as rigidity and perfectionism, creating a self-perpetuating trait–state interaction loop. Biological alterations reinforce restrictive behavior, which in turn sustains biological dysregulation.

### 4.3. Personality and Relational Context

Biological mechanisms alone do not determine symptom selection. Eating disorders unfold within specific relational environments. Many patients exhibit high self-criticism, fear of dependency, and attachment insecurity. In certain family systems characterized by control or emotional enmeshment, the symptom may regulate systemic tension [15].

The integration of personality structure and systemic dynamics renders linear causal models insufficient. Behavioral interventions may achieve short-term symptom reduction; however, without addressing relational and identity-level functions, changes often remain fragile.

Thus, a comprehensive model must integrate biological predisposition, starvation-induced neuroadaptations, personality organization, and systemic processes. This integration sets the stage for understanding why somatic stabilization is both medically urgent and psychologically complex.

## 5. Anorexia in Life-Threatening Stages: Somatic Safety and Psychological Cost

Given the high mortality of anorexia nervosa [6], somatic stabilization constitutes the primary clinical priority. Hospitalization, nutritional rehabilitation, and structured monitoring are evidence-based necessities in severe cases [18,27]. In such contexts, temporary limitation of autonomy is ethically justified.

However, these interventions carry psychological cost. In anorexia, control overeating often represents the core of identity and perceived safety. Removing this control may activate shame, anger, and fear of dependency. Thus, life-saving procedures are never psychologically neutral.

Treatment settings must therefore balance medical rigor with relational sensitivity. Protocols function either as therapeutic scaffolding or as perceived coercion, depending on communication and alliance. When experienced as “auxiliary ego” support, structured interventions can facilitate stabilization; when perceived as punitive control, they may intensify resistance.

As weight restoration progresses, psychotherapeutic work becomes possible. Here, rigid application of weighing protocols warrants reconsideration. While weight remains an objective safety marker, excessive focus may reinforce obsessive monitoring. Flexible approaches—including blind weighing—may preserve alliance while maintaining medical oversight.

Similarly, body-image interventions must extend beyond perceptual correction. The body in EDs is symbolically saturated—with shame, aggression, dependency, and control. Behavioral techniques achieve structural change only when embedded in relational and affective work.

Thus, early treatment stages illustrate the central thesis of this article: symptom-focused intervention is necessary but insufficient. Behavioral stabilization must gradually transition into integrative work addressing trait vulnerabilities and starvation-induced adaptations within relational contexts.

## 6. The Role of Unconscious Processes in the Treatment of Eating Disorders

While CBT-based approaches focus primarily on conscious beliefs and behavioral modification, clinical work with patients with eating disorders often reveals processes that exceed purely cognitive formulations. Focusing exclusively on conscious distortions may overlook deeper motivational and relational processes.

Importantly, Aaron Beck did not conceptualize cognition as isolated from deeper psychological processes. Early theoretical writings [28,29] suggest clear continuity with psychodynamic thinking, particularly in the recognition that preconscious and implicit structures organize conscious experience. Beck’s later theoretical development [4,5] retained the assumption that enduring schemas shape perception, affect, and behavior beyond immediate awareness. Similarly, Kihlstrom [30,31] described unconscious processes as organized emotional–cognitive systems influencing behavior outside deliberate control.

In the context of eating disorders, this perspective becomes clinically salient. Patients frequently articulate a conscious desire to recover while simultaneously engaging in behaviors that undermine recovery. Statements such as “I want to get better, but I cannot gain weight” reflect a structural ambivalence. The symptom may be experienced as both intolerable and indispensable.

Such ambivalence cannot be fully explained through automatic thoughts alone. The eating symptom may serve unconscious regulatory functions: defending against dependency, shielding from overwhelming affect, preserving identity coherence, or maintaining relational equilibrium [32,33]. Resistance to change may therefore represent not mere cognitive avoidance but an attempt to preserve psychological organization under perceived threat.

From a clinical standpoint, addressing unconscious processes does not negate behavioral intervention. Rather, it reframes resistance as meaningful. The therapeutic task includes identifying the regulatory function of restriction or binge–purge behaviors within the patient’s internal and relational world. Premature dismantling of the symptom—without providing alternative regulatory structures—may destabilize fragile psychological equilibrium.

Thus, an integrative approach situates CBT techniques within a broader developmental and relational framework. Engaging unconscious dynamics strengthens—not weakens—the potential for sustainable behavioral change. This perspective prepares the ground for examining empirical limitations of protocol-driven models.

## 7. Empirical Limitations of Contemporary CBT-ED Protocols

CBT-ED developed within a scientific context emphasizing measurability, manualization, and replicability. These strengths enabled the establishment of structured treatment protocols and facilitated empirical validation. However, the same characteristics that ensure methodological clarity may limit clinical flexibility.

Evidence indicates that CBT-ED demonstrates robust efficacy in bulimia nervosa and binge-eating disorder, but more modest and less stable outcomes in anorexia nervosa [34]. Remission rates in AN remain comparatively low, and relapse rates substantial. Linardon et al. [27,35] highlight heterogeneity across trials and limited long-term follow-up data. Dobson et al. [12] further note that cognitive–behavioral approaches show diminished effectiveness in patients presenting with pronounced personality rigidity, trauma exposure, or chronicity.

These findings do not undermine CBT as a first-line intervention; rather, they delineate its boundaries. In anorexia nervosa especially, starvation-induced neurobiological adaptations may impair cognitive flexibility and executive functioning, reducing responsiveness to purely cognitive restructuring. Without concurrent nutritional rehabilitation, cognitive work may confront a biologically constrained system.

Moreover, the procedural structure of CBT may be absorbed into pre-existing perfectionistic and overcontrolled personality styles. Highly compliant patients may excel at completing worksheets while maintaining core regulatory patterns unchanged. In such cases, therapy risks reinforcing control rather than transforming it.

Vogel et al. [36] suggest that family-based therapy may outperform CBT in adolescents, underscoring the systemic dimension of maintenance mechanisms. Similarly, research on treatment refusal and premature termination [37] indicates that rigid application of protocols may contribute to disengagement in certain subgroups.

Taken together, empirical findings suggest that CBT-ED is necessary but not universally sufficient. Its effectiveness may depend on integration with nutritional, relational, and developmental interventions addressing both trait vulnerabilities and starvation-induced state effects.

These limitations become particularly visible in discussions surrounding the weighing procedure in CBT-E.

## 8. Conclusions from Scandinavian Research and Reviews

Scandinavian researchers have offered a critical evaluation of cognitive models of eating disorders [38,39]. Södersten and colleagues argue that multi-component cognitive formulations—emphasizing overvaluation of weight and shape, perfectionism, and distress intolerance—may lack clear causal hierarchy. According to their critique, circular reasoning may emerge when beliefs are treated simultaneously as causes and consequences of behavior.

Importantly, they propose that many cognitive and affective symptoms conceptualized as “core psychopathology” may represent starvation-induced adaptations rather than primary etiological mechanisms. Emotional dysregulation, rigidity, and preoccupation with food may partially reflect malnutrition-driven neurobiological changes.

This argument aligns with emerging neurobiological models emphasizing trait–state interaction. If starvation itself intensifies rigidity and anxiety, then cognitive distortions cannot be interpreted independently of metabolic status. Behavioral normalization—particularly structured eating—may, therefore, exert therapeutic effects not solely through cognitive change but via neurobiological recalibration.

Reported remission rates below 50% and relapse rates exceeding 30% within one year further highlight the need to clarify mechanisms of change [38]. Improvement may be attributable primarily to behavioral normalization rather than cognitive restructuring per se.

These critiques do not reject CBT but advocate refinement. Distinguishing trait-based vulnerability from starvation-induced adaptation becomes essential for identifying primary therapeutic targets. This distinction is associated with reconsideration of specific CBT-E techniques.

## 9. Critical Analysis of the Fairburn Protocol (CBT-E)

Within CBT-E, regular weighing is conceptualized as exposure designed to reduce anxiety through habituation [1,2]. The underlying assumption is that repeated contact with weight measurements decreases catastrophic interpretation and facilitates cognitive restructuring.

Clinically, however, weight rarely functions as a neutral stimulus in anorexia nervosa. It represents control, moral worth, identity, and relational positioning. Consequently, weighing may not extinguish anxiety but intensify anticipatory arousal and ritualized monitoring.

Empirical findings provide mixed support for universal application of this technique. Swift et al. [37] report increased treatment dropout associated with distress during exposure-based procedures. Engel et al. [14] demonstrate the centrality of affect regulation in symptom maintenance. Italiano et al. [40] describe trauma-related responses in subgroups exposed to highly activating interventions. Waller and Mountford [41] note that rigid weighing protocols may exacerbate relapse risk in vulnerable individuals.

Alternative approaches emphasizing structured eating without mandatory weighing show comparable clinical outcomes with reduced anxiety in some studies [42]. These findings suggest that weighing may require individualized implementation rather than strict standardization.

From a trait–state perspective, frequent weighing may amplify starvation-induced cognitive rigidity and perfectionism. When leptin levels remain low and HPA activation elevated, exposure to weight fluctuations may reinforce threat processing rather than habituation.

Furthermore, in certain family contexts, weight serves as a symbolic site of control and negotiation. Repeated measurement may inadvertently stabilize maladaptive systemic dynamics.

Therefore, the question is not whether CBT-E should be abandoned, but how it can be integrated into a broader framework that accounts for neurobiological maintenance, relational meaning, and trait vulnerability. Structured behavioral tools remain essential for safety; however, their application must be developmentally and biologically informed.

This critical analysis prepares the transition to the next section. If starvation-induced adaptations contribute substantially to maintenance mechanisms, then nutritional rehabilitation must be conceptualized not merely as supportive care but as a primary neurobiological intervention.

## 10. Nutritional Rehabilitation as Neurobiological Recalibration

Within the trait–state framework outlined above, nutritional rehabilitation should be conceptualized not merely as supportive care or weight restoration, but as a primary form of neurobiological recalibration. If starvation-induced adaptations actively sustain eating disorder psychopathology, then restoration of adequate energy and micronutrient intake becomes a prerequisite for reversing the biological processes that perpetuate symptoms [17].

Refeeding promotes normalization of metabolic and endocrine functioning, including partial restoration of leptin signaling, recovery of hypothalamic–pituitary–gonadal axis activity, and modulation of stress responsivity. Chronic hyperactivation of the hypothalamic–pituitary–adrenal (HPA) axis and sustained hypercortisolemia are well-documented features of anorexia nervosa, particularly among adolescents [10,22]. Progressive weight restoration has been associated with improvements in leptin levels and broader endocrine regulation [11], which may reduce hyperarousal, anticipatory anxiety, and cognitive slowing.

Importantly, serotonergic modulation appears directly linked to affective regulation in anorexia nervosa. In a controlled experimental study, Kaye et al. demonstrated that acute tryptophan depletion produced transient anxiolytic effects in individuals recovered from anorexia nervosa [24]. Subsequent neuroimaging studies showed that acute tryptophan depletion modulates resting-state connectivity within the salience network [9] and alters instrumental reward learning processes [43]. Together, these findings support the hypothesis that dietary restriction—via reduced tryptophan availability and decreased central serotonin synthesis—may function as a biologically reinforced affect-regulation strategy.

Refeeding interrupts this mechanism. By restoring serotonergic precursors and stabilizing neurotransmitter availability, nutritional rehabilitation weakens starvation-driven anxiolytic reinforcement and reduces the neurochemical incentives maintaining restriction.

Beyond monoaminergic systems, severe anorexia nervosa is associated with high rates of micronutrient deficiencies, including zinc, iron, selenium, and B vitamins [44,45]. These deficits are linked to fatigue, irritability, impaired executive functioning, and depressive symptomatology. Polyunsaturated fatty acid (PUFA) alterations have also been associated with eating disorder psychopathology [46], suggesting possible involvement of inflammatory and membrane-level regulatory processes.

Appetite-regulating hormones further contribute to neurobiological dysregulation. Hypoleptinemia is characteristic of acute anorexia nervosa [11], while altered ghrelin signaling—including emerging data on LEAP2—has been implicated in disrupted hunger processing and motivational salience attribution. Restoration of energy balance may recalibrate interoceptive signaling and reward sensitivity, thereby improving decision-making under conditions of weight restoration.

Longitudinal data suggest that weight restoration is associated with measurable changes in brain structure and functional connectivity, including partial recovery of gray matter volume and network integration. Such findings reinforce the concept that nutritional rehabilitation operates at both metabolic and neural levels.

Crucially, improvement in executive functioning and cognitive flexibility following refeeding may enhance patients’ capacity to engage in psychotherapeutic interventions. In this sense, nutritional rehabilitation and psychotherapy should not be viewed as sequential steps, but as biologically complementary processes.

These observations support a broader trait–state interaction model, in which starvation-induced neurobiological adaptations interact with pre-existing vulnerabilities to sustain symptom persistence and relapse risk. This model is elaborated in the following section.

However, this finding requires careful interpretation. Existing studies are based on relatively small samples and often involve individuals in specific stages of illness or recovery. The anxiolytic effect of acute tryptophan depletion may not generalize across populations and should not be interpreted as evidence that dietary restriction is therapeutically beneficial. Alternative explanations—including context-dependent neuroadaptation and individual variability in serotonergic functioning—must be considered. From a clinical perspective, misinterpretation of these findings could be problematic and underscores the need for cautious framing.

These findings are derived from multiple methodologies, including neuroimaging techniques (e.g., PET, fMRI), endocrine assessments, and pharmacological challenge studies, and vary depending on nutritional state and stage of illness.

## 11. Transdiagnostic Biological Processes

The mechanisms described above provide the basis for understanding nutritional rehabilitation as a process of neurobiological recalibration. Accumulating evidence indicates that eating disorders (EDs) should not be conceptualized solely as disturbances of behavior or cognition, but rather as complex conditions emerging from dynamic interactions between trait-based vulnerabilities and state-dependent neurobiological adaptations induced by malnutrition. Although maladaptive beliefs concerning weight, shape, and control constitute central diagnostic features, increasing data suggest that these cognitive patterns are embedded within broader biological processes that both predispose individuals to illness and are subsequently amplified by starvation-induced adaptations [21,22,23].

This trait–state interaction model provides a coherent framework for understanding symptom persistence, diagnostic crossover, and relapse vulnerability across ED phenotypes.

### 11.1. Trait–State Interaction Framework

Trait-based vulnerabilities include heightened anxiety, behavioral inhibition, perfectionism, cognitive rigidity, and intolerance of uncertainty, often accompanied by alterations in serotonergic and dopaminergic signaling. Neuroimaging and pharmacological challenge studies suggest that some of these features may reflect enduring neurobiological characteristics rather than purely psychological constructs [21,23].

However, once dietary restriction begins, starvation triggers a cascade of metabolic, endocrine, and neurotransmitter changes. These state-dependent adaptations intensify pre-existing vulnerabilities and generate self-reinforcing feedback loops.

Thus, eating disorders are maintained not only by dysfunctional cognitions but also by biologically mediated reinforcement processes in which malnutrition itself perpetuates the disorder.

### 11.2. Serotonergic Dysregulation as a Transdiagnostic Mechanism

One of the most consistently described mechanisms involves serotonergic dysregulation.

Individuals predisposed to restrictive phenotypes frequently exhibit:

- Heightened anxiety;
- Harm avoidance;
- Cognitive rigidity;
- Behavioral overcontrol.

These traits have been associated with alterations in serotonin receptor binding and serotonergic tone [21,23].

Importantly, dietary restriction reduces tryptophan availability, thereby lowering central serotonin synthesis. Experimental evidence demonstrates that acute tryptophan depletion may transiently reduce anxiety in individuals with current or recovered anorexia nervosa [24]. Neuroimaging findings further show modulation of salience-network connectivity and reward learning under tryptophan depletion conditions [9,43].

This paradoxical anxiolytic effect suggests that starvation may function as a form of maladaptive neurochemical self-regulation. Reduced serotonergic activity may temporarily attenuate hyperserotonergic states associated with anxiety and overcontrol, thereby negatively reinforcing restrictive behavior.

This mechanism extends beyond anorexia nervosa. Serotonergic dysregulation is also implicated in obsessive–compulsive disorder and anxiety disorders, suggesting a shared vulnerability dimension expressed behaviorally in different ways across diagnostic categories [21].

### 11.3. Dopaminergic Reward Processing and Reinforcement Learning

Dopaminergic dysfunction represents another central transdiagnostic pathway.

In anorexia nervosa, neuroimaging studies demonstrate:

- Reduced reward responsiveness to food stimuli;
- Heightened motivational salience attributed to weight loss, control, or excessive exercise;
- Altered temporal-difference learning processes [25,26].

This reversal of reward valuation may explain the persistence of restrictive behaviors despite physiological deprivation.

Conversely, binge-eating disorder and impulsive phenotypes often display:

- Heightened cue reactivity;
- Altered reinforcement learning;
- Impaired inhibitory control.

Despite divergent behavioral expressions, both restrictive and binge–purge presentations involve dysregulated valuation processes within mesolimbic circuits.

Thus, dopaminergic alterations may represent a shared neurobiological substrate expressed through either overcontrol or impulsivity depending on individual trait configuration and environmental context.

### 11.4. HPA Axis Hyperactivation and Stress Sensitivity

Chronic activation of the hypothalamic–pituitary–adrenal (HPA) axis is a well-documented feature of restrictive eating disorders [22]. Sustained hypercortisolemia contributes to:

- Hypervigilance;
- Anticipatory anxiety;
- Impaired stress extinction;
- Cognitive inflexibility.

Importantly, elevated cortisol levels may further impair prefrontal functioning and reinforce rigid decision-making under stress.

HPA axis dysregulation is not specific to EDs; it is also observed in major depressive disorder and trauma-related conditions. This overlap supports the notion that EDs share stress-related neurobiological vulnerabilities with other psychiatric disorders.

Within the trait–state framework, starvation-induced HPA activation amplifies pre-existing anxiety sensitivity, thereby intensifying restrictive behavior as a perceived regulatory strategy.

### 11.5. Appetite-Regulating Hormones: Leptin and Ghrelin

Metabolic hormones play a critical role in the maintenance of ED psychopathology.

In anorexia nervosa, marked hypoleptinemia reflects chronic energy insufficiency [11]. Beyond its homeostatic role, leptin modulates:

- Hypothalamic circuits;
- Mesolimbic reward pathways;
- Executive control regions.

Persistently low leptin concentrations have been associated with hyperactivity, food preoccupation, and reproductive dysfunction. During refeeding, increasing leptin levels may signal partial neuroendocrine recalibration; however, rapid increases may also coincide with heightened anxiety in some individuals.

Conversely, binge-eating disorder frequently involves elevated leptin levels, potentially indicating central leptin resistance and impaired satiety signaling.

Ghrelin, an orexigenic hormone involved in hunger signaling and reward modulation, is paradoxically elevated in anorexia nervosa despite chronic restriction [47]. This phenomenon has led to the hypothesis of ghrelin resistance, in which increased circulating levels fail to produce adaptive feeding behavior.

Because ghrelin influences dopaminergic activity within mesolimbic pathways, altered ghrelin signaling may affect motivational salience and reinforcement learning.

Incomplete normalization of leptin and ghrelin signaling after weight restoration may contribute to relapse vulnerability by sustaining altered reward processing and interoceptive uncertainty.

### 11.6. Micronutrient Deficiencies and Neurocognitive Function

Micronutrient deficiencies represent an often underestimated biological dimension of EDs.

Severely malnourished individuals frequently present with deficiencies in:

- Zinc;
- Iron;
- Selenium;
- Vitamin D;
- B-complex vitamins [44,45].

Micronutrients function as essential cofactors in neurotransmitter synthesis (particularly serotonin and dopamine), mitochondrial energy production, and antioxidant defense systems. Deficiencies in zinc, iron, selenium, and B-complex vitamins may contribute to fatigue, irritability, executive dysfunction, and impaired stress tolerance, thereby reinforcing maladaptive coping strategies centered around restriction or dysregulated eating. Importantly, these deficiencies are not trait vulnerabilities but state-dependent consequences of starvation that amplify pre-existing psychological and neurobiological patterns. To facilitate comparison across mechanisms and levels of evidence, Table 1 summarizes key neurobiological and nutritional processes in eating disorders, including diagnosis, nutritional state, and main limitations of the available data.

However, the strength of evidence varies substantially across micronutrients, ranging from observational prevalence data to limited mechanistic and interventional studies.

### 11.7. Integrated Trait–State Model (Linked to Table 2 and Figure 1)

Table 2 conceptualizes EDs within a structured trait–state matrix, distinguishing baseline vulnerabilities from starvation-induced amplifications.

**Figure 1:**
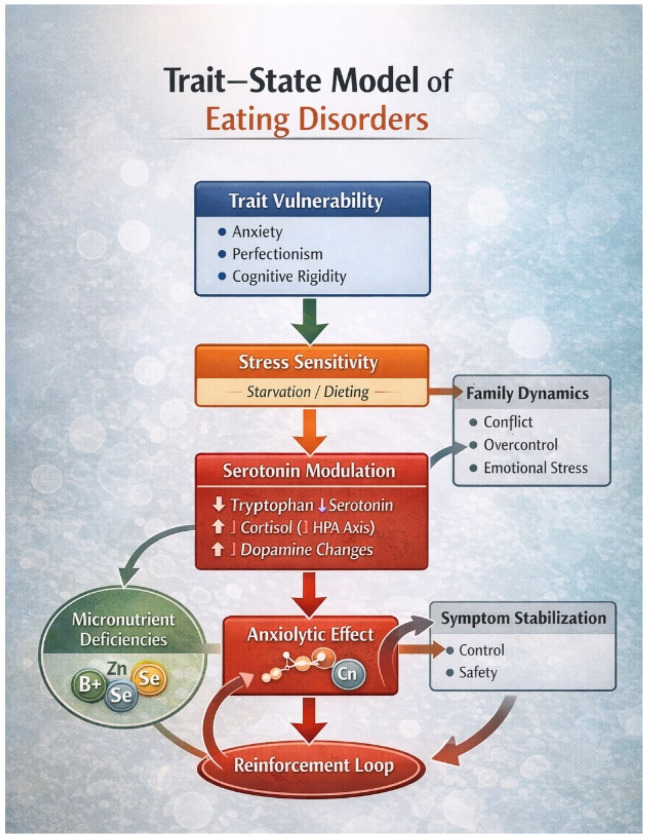
Trait–State Transdiagnostic Model of Eating Disorders. Predisposing trait vulnerabilities (e.g., anxiety, perfectionism, cognitive rigidity) increase stress sensitivity and contribute to food restriction. Dietary restriction induces neurobiological modulation, including altered serotonergic signaling, HPA axis activation, and dopaminergic changes. These adaptations may produce transient anxiolytic effects, reinforcing restrictive behavior and creating a self-perpetuating loop. Family dynamics may further stabilize symptom expression by modulating stress and control-related processes. Figure created by the authors.

Figure 1 illustrates the cyclical feedback loop:

1. Trait vulnerabilities (e.g., anxiety, perfectionism, rigidity);
2. Initiation of dietary restriction;
3. Starvation-induced neurobiological adaptations;
4. Transient anxiolytic or regulatory effects;
5. Reinforcement of restrictive behavior.

Family and relational dynamics may further stabilize this loop by modulating stress, control, and attachment processes.

Together, these interacting mechanisms generate a self-perpetuating cycle in which biological recalibration through nutritional rehabilitation becomes a prerequisite for sustainable psychological change.

**Table 2:** Trait–State Transdiagnostic Model in Anorexia Nervosa.

Trait-Based Vulnerability	Baseline Characteristic	State-Dependent Neurobiological and Psychological Alterations
Temperament/personality structure	A personality profile characterized by high trait anxiety, behavioral inhibition, perfectionism, overcontrol, cognitive rigidity, and pronounced self-criticism	An intensification of cognitive rigidity, increased perseverative tendencies, diminished decisional flexibility, and escalation of compulsive and ritualized behaviors
Emotion regulation	An emotion regulation profile marked by impaired affect tolerance, intolerance of uncertainty, a tendency toward emotional avoidance, and excessive regulation or suppression of emotional states	Heightened irritability, affective instability, diminished mentalizing capacity, and increased anxiety-driven and affective reactivity
Cognitive functions	A cognitive style characterized by dichotomous (all-or-nothing) thinking and rigid, perfectionistic standards of self-evaluation	Psychomotor and cognitive slowing, reduced concentration, deterioration of executive functioning, and narrowing of attentional scope
Schemas and core beliefs	Early maladaptive schemas involving defectiveness/unworthiness, abandonment, unrelenting standards, dependence, and pervasive shame	Consolidation of control-related core beliefs and catastrophic interpretations of eating and weight restoration
Relationships and attachment	Attachment patterns marked by fear of dependency, ambivalence toward intimacy, and difficulties in the separation–individuation process	Escalation of social withdrawal, heightened interpersonal sensitivity and irritability, and increased suspiciousness in relational contexts
The function of the symptom	A psychological need to maintain control, regulate affect, and preserve a stable sense of identity and relational equilibrium	Neurobiological entrenchment of the symptom, whereby starvation acquires a self-regulatory neurochemical function
The serotonergic system	A biological predisposition toward hyperserotonergic activity, linked to heightened trait anxiety and increased behavioral inhibition	Dietary depletion of tryptophan is associated with relative reduction in serotonergic activity, which may be experienced as decreased anxiety and subjective relief, thereby biologically reinforcing dietary restriction as a strategy of affect regulation
The reward system (dopaminergic pathways)	Atypical reward processing characterized by diminished reactivity to hedonic stimuli and heightened sensitivity to control- and avoidance-based reinforcement	Paradoxical reinforcement of dietary restriction and hyperactivity, accompanied by anxiety—rather than hedonic reward—in response to food-related stimuli
The hypothalamic–pituitary–adrenal (HPA) axis	Increased stress reactivity, persistent hypervigilance, and impaired extinction of stress responses, consistent with dysregulation of the HPA axis	Sustained hypercortisolemia and chronic HPA-axis activation, associated with heightened hypervigilance, anticipatory anxiety, and diminished tolerance for frustration
Appetite-regulating hormones	Potential biological vulnerabilities in appetite regulation and altered processing of interoceptive (bodily) signals	Hypoleptinemia and hyperghrelinemia, accompanied by impaired responsiveness to hunger cues and dysregulation of eating-related decision-making processes
Micronutrients	None—not regarded as baseline characteristics	Micronutrient deficiencies (zinc, iron, selenium, B-complex vitamins, and vitamin D) associated with fatigue, increased irritability, and impaired cognitive performance and mood regulation

### 11.8. Implications for a Transdiagnostic Framework

The convergence of serotonergic, dopaminergic, endocrine, and metabolic alterations across ED phenotypes supports a dimensional rather than purely categorical understanding.

Within this model:

- Trait vulnerabilities predispose individuals to specific regulatory strategies.
- Starvation-induced adaptations biologically reinforce those strategies.
- Hormonal and micronutrient alterations modulate cognitive flexibility and stress sensitivity.
- Neurobiological recalibration becomes central to relapse prevention.

Thus, EDs may be conceptualized as biologically mediated regulatory disorders in which trait-based predispositions interact dynamically with state-dependent adaptations.

This framework provides a mechanistic bridge between Section 9 and Section 11, linking nutritional rehabilitation as neurobiological recalibration with broader transdiagnostic relevance across psychiatric conditions.

### 11.9. Differences Across Eating Disorder Diagnoses

Although the mechanisms described above are presented within a transdiagnostic framework, it is important to note that a substantial proportion of neurobiological evidence derives from studies of anorexia nervosa, particularly in underweight states.

In anorexia nervosa, starvation-related mechanisms—including hypoleptinemia, hypercortisolemia, and reduced tryptophan availability—appear to play a central role in symptom maintenance. In contrast, bulimia nervosa and binge-eating disorder are more often characterized by fluctuating energy balance, patterns of restriction and binging, and alterations in reward sensitivity and impulse control.

Dopaminergic reward processing may manifest differently across diagnoses: restrictive phenotypes are associated with reduced reward responsiveness to food and increased salience of control-based behaviors, whereas binge-eating presentations often involve heightened cue reactivity and impaired inhibitory control.

These differences suggest that some mechanisms are primarily starvation-driven (state-dependent), while others reflect broader trait-based vulnerabilities that may be expressed across diagnostic categories irrespective of weight status.

## 12. Transdiagnostic Relevance Beyond Anorexia Nervosa

Although the neurobiological model outlined in the preceding sections has historically been centered on anorexia nervosa (AN), increasing empirical evidence supports its broader, transdiagnostic relevance. The trait–state interaction framework described in Section 10 is not limited to restrictive phenotypes but appears to reflect more general mechanisms of psychopathology that cut across diagnostic categories.

### 12.1. Serotonergic Vulnerability Across Disorders

Serotonergic dysregulation, strongly implicated in AN, has been associated with heightened anxiety, harm avoidance, and cognitive rigidity—traits frequently present prior to illness onset and persisting after weight restoration [21,23]. These characteristics overlap substantially with obsessive–compulsive disorder (OCD) and anxiety disorders, where altered serotonergic functioning has likewise been documented through pharmacological and neuroimaging research.

Within the trait–state model, serotonergic vulnerability represents a trait-based predisposition that may manifest behaviorally in different ways depending on environmental context and regulatory strategies. In AN, dietary restriction may transiently attenuate hyperserotonergic states through tryptophan depletion, producing short-term anxiolytic effects [24]. In OCD, similar serotonergic profiles may instead manifest through compulsive rituals unrelated to food. Thus, restrictive eating can be conceptualized as one possible regulatory strategy within a broader serotonergic vulnerability spectrum.

### 12.2. Dopaminergic Reward Dysregulation as a Shared Mechanism

Alterations in dopaminergic reward processing constitute another transdiagnostic pathway. In AN, neuroimaging findings indicate reduced reward responsiveness to food stimuli and paradoxical reinforcement associated with weight loss, excessive exercise, or control-based behaviors [25,26]. These findings align with models emphasizing altered valuation and reinforcement learning.

In contrast, binge-eating disorder (BED) and substance-use disorders are frequently characterized by heightened cue reactivity and impulsive reward-seeking behavior. Despite these behavioral differences, both restrictive and binge–purge presentations involve dysregulated mesolimbic circuitry and impaired integration of reward signals with executive control systems.

Within the trait–state framework, pre-existing differences in reward sensitivity and inhibitory control may determine whether dopaminergic dysregulation manifests as restrictive overcontrol or impulsive overeating. Starvation-induced adaptations further modify reward valuation, reinforcing maladaptive patterns and contributing to diagnostic crossover.

### 12.3. HPA Axis Hyperactivation and Stress Sensitivity

Chronic activation of the hypothalamic–pituitary–adrenal (HPA) axis is a consistent finding in AN, with elevated cortisol levels reflecting sustained stress responsivity [10,22]. However, HPA axis dysregulation is also central to major depressive disorder and post-traumatic stress disorder.

In the context of eating disorders, persistent hypercortisolemia may amplify threat sensitivity, intolerance of uncertainty, and cognitive inflexibility. Starvation-induced adaptations may therefore intensify pre-existing stress reactivity, creating a feedback loop in which physiological stress responses reinforce restrictive or compensatory behaviors.

Importantly, this suggests that stress-related neuroendocrine vulnerability is not disorder-specific but represents a shared biological substrate across multiple psychiatric conditions.

### 12.4. Perfectionism and Cognitive Rigidity as Cross-Diagnostic Traits

Perfectionism, self-criticism, and intolerance of uncertainty are widely documented in AN and other eating disorders. However, these traits are also prevalent in anxiety disorders, OCD, and depression. Neurocognitive research links such traits to heightened error monitoring and rigid control processes, potentially mediated by frontostriatal circuitry.

Within the trait–state model, perfectionism functions as a trait-based vulnerability that may become amplified by starvation-induced cognitive narrowing and executive dysfunction. Malnutrition reduces cognitive flexibility, increases perseverative tendencies, and strengthens dichotomous thinking, thereby biologically reinforcing rigid personality traits that predated illness onset.

### 12.5. Micronutrient Deficiencies and Broader Psychiatric Relevance

Micronutrient deficiencies—particularly involving iron, zinc, selenium, vitamin D, and B-complex vitamins—have been documented in severely malnourished individuals with AN [44,45]. These deficiencies are associated with fatigue, impaired executive functioning, irritability, and depressive symptoms.

Beyond eating disorders, micronutrient status has been linked to broader psychiatric outcomes, including mood and cognitive disturbances. Given their role as cofactors in neurotransmitter synthesis and mitochondrial energy metabolism, micronutrients represent a biologically plausible pathway connecting nutritional state to psychiatric symptom expression.

### 12.6. Dimensional Implications

Taken together, these findings support a dimensional model in which shared neurobiological vulnerabilities—serotonergic sensitivity, dopaminergic reward dysregulation, HPA axis hyperactivation, and impaired interoceptive signaling—manifest across diagnostic categories. Eating disorders may therefore represent one clinically distinct configuration of overlapping mechanisms rather than an isolated nosological entity.

This transdiagnostic perspective strengthens the argument that starvation-induced adaptations should not be conceptualized solely as consequences of eating pathology, but as powerful modulators of broader neurobiological systems involved in anxiety, reward, stress, and executive control.

## 13. Conclusions

The evidence synthesized in this article supports a multifactorial and transdiagnostic understanding of eating disorders grounded in dynamic interactions between trait-based vulnerabilities and starvation-induced neurobiological adaptations.

### 13.1. Trait–State Interaction and Maintenance of Symptoms

Trait vulnerabilities—including heightened anxiety sensitivity, perfectionism, behavioral inhibition, and altered reward processing—create a neurobiological and psychological substrate that increases susceptibility to restrictive or dysregulated eating patterns. Once dietary restriction or binge–purge cycles emerge, starvation-induced adaptations further modify serotonergic, dopaminergic, and endocrine functioning.

These state-dependent alterations reshape affect regulation, stress responsivity, cognitive flexibility, and reward valuation. Reduced tryptophan availability may transiently attenuate hyperserotonergic states [24], reinforcing restriction as an affect-regulation strategy. Dopaminergic shifts may recalibrate reward salience toward control-based behaviors [25,26]. HPA axis hyperactivation sustains hypervigilance and anticipatory anxiety [10,22]. Appetite-regulating hormones such as leptin and ghrelin influence motivational and interoceptive processes, contributing to persistent dysregulation [11,47].

In this framework, restrictive or dysregulated eating behaviors become biologically reinforced regulatory strategies rather than merely products of distorted cognition.

### 13.2. Nutritional Rehabilitation as Neurobiological Recalibration

Conceptualizing nutritional rehabilitation as neurobiological recalibration—rather than simple weight restoration—has significant therapeutic implications. Adequate energy intake and correction of micronutrient deficiencies are likely prerequisites for restoring cognitive flexibility, reducing hyperarousal, and weakening starvation-driven reinforcement loops.

Refeeding contributes to normalization of endocrine parameters, including leptin signaling [11], and may attenuate chronic HPA axis activation [10]. Correction of micronutrient deficiencies [44,45] supports neurotransmitter synthesis and executive functioning. Together, these processes enhance patients’ capacity to engage in psychotherapeutic work.

Thus, nutritional and psychological interventions should be conceptualized as biologically complementary rather than sequential. Psychotherapy may be limited in effectiveness when delivered in the context of ongoing starvation-induced cognitive rigidity and neurochemical imbalance.

### 13.3. Clinical Integration

An integrative treatment framework emerges from this synthesis. Behavioral stabilization and medical safety remain essential. However, long-term recovery likely requires addressing:

- Trait-based vulnerabilities (e.g., perfectionism, intolerance of uncertainty);
- Starvation-induced neurobiological adaptations;
- Relational and systemic dynamics that stabilize symptom expression.

Recognition of biologically mediated reinforcement loops may reduce misinterpretation of resistance as mere noncompliance. Instead, reluctance to change may reflect neurobiologically entrenched regulatory mechanisms requiring gradual recalibration.

### 13.4. Future Directions

Future research should prioritize longitudinal and multimodal designs integrating:

- Neuroimaging;
- Endocrine markers;
- Micronutrient assessment;
- Detailed clinical phenotyping.

**Figure 2:**
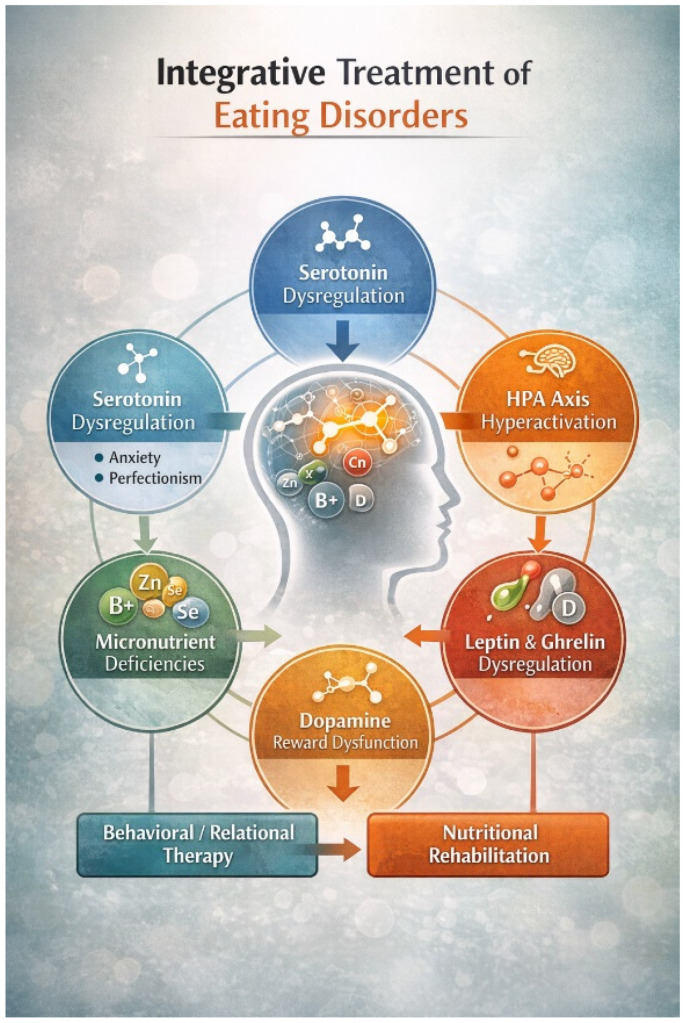
Integrative neurobiological framework for treatment of eating disorders. Eating disorders involve interacting biological mechanisms, including serotonergic dysregulation, dopaminergic reward alterations, HPA axis hyperactivation, appetite-hormone imbalance (leptin and ghrelin), and micronutrient deficiencies. Nutritional rehabilitation and behavioral/relational interventions act on complementary levels: restoring metabolic and neurochemical balance while addressing trait vulnerabilities and maladaptive regulatory patterns. This integrative approach conceptualizes treatment as both behavioral stabilization and neurobiological recalibration. Figure created by the authors.

Such approaches may clarify the relative contributions of trait predispositions and state-dependent adaptations, identify biomarkers of relapse risk, and refine individualized treatment planning (Figure 2).

These findings highlight the importance of integrating nutritional rehabilitation early in treatment as a necessary condition for restoring cognitive flexibility, emotional regulation, and responsiveness to psychotherapeutic interventions.

These findings should be interpreted with consideration of the heterogeneity of study designs and potential confounding effects of malnutrition and comorbidity.

## Figures and Tables

**Table 1:** Summary of neurobiological and nutritional mechanisms across eating disorders.

Mechanism	Diagnosis	Nutritional State	Evidence Type	Main Finding	Limitations	Key References
Serotonergic dysregulation	AN (mainly)	Underweight/recovered	PET, depletion studies	Altered serotonin linked to anxiety and restriction reinforcement	Small samples, heterogeneity	[21,23,24]
Dopaminergic reward alterations	AN, BN, BED	Mixed	fMRI	Altered reward valuation (food vs. control)	Task variability	[25,26]
HPA axis activation	AN	Underweight	Endocrine studies	Elevated cortisol, stress sensitivity	Confounded by malnutrition	[10,22]
Leptin dysregulation	AN	Underweight	Hormonal studies	Hypoleptinemia linked to hyperactivity, cognition	Normalization varies	[11]
Ghrelin alterations	AN, BED	Mixed	Hormonal studies	Dysregulated hunger signaling	Possible resistance mechanisms	[47]
Micronutrient deficiencies	AN	Underweight	Clinical studies	Cognitive and mood effects	Mostly observational	[44,45]

## Data Availability

No new data were created or analyzed in this study.
